# Association of Digital Engagement With Relaxation Tools and Stress Level Reduction: Retrospective Cohort Study

**DOI:** 10.2196/50506

**Published:** 2024-03-19

**Authors:** Inbar Breuer-Asher, Marilyn D Ritholz, David L Horwitz, Omar Manejwala, Ephraim Behar, Yifat Fundoiano-Hershcovitz

**Affiliations:** 1 DarioHealth Caesarea Israel; 2 Joslin Diabetes Center Harvard Medical School Boston, MA United States; 3 DLH Biomedical Consulting Las Vegas, NV United States

**Keywords:** mental health, perceived stress, stress reduction, digital health, video sessions, behavioral health, relaxation, breathing exercises, CBT, anxiety, cognitive behavioral therapy

## Abstract

**Background:**

Stress is an emotional response caused by external triggers and is a high-prevalence global problem affecting mental and physical health. Several different digital therapeutic solutions are effective for stress management. However, there is limited understanding of the association between relaxation components and stress levels when using a digital app.

**Objective:**

This study investigated the contribution of relaxation tools to stress levels over time. We hypothesized that participation in breathing exercises and cognitive behavioral therapy–based video sessions would be associated with a reduction in stress levels. We also hypothesized a significant reduction specifically in participants’ perceived sense of burden and lack of productivity when engaged with breathing exercises and video sessions.

**Methods:**

Stress levels were evaluated in a real-world data cohort using a behavioral health app for digital intervention and monitoring change. This retrospective real-world analysis of users on a mobile platform–based treatment followed users (N=490) who started with moderate and above levels of stress and completed at least 2 stress assessments. The levels of stress were tracked throughout the first 10 weeks. A piecewise mixed effects model was applied to model the trajectories of weekly stress mean scores in 2 time segments (1-6 weeks and 6-10 weeks). Next, a simple slope analysis was used for interpreting interactions probing the moderators: breathing exercises and video sessions. Piecewise mixed-effects models were also used to model the trajectories of specific perceived stress item rates in the stress questionnaire in the 2 segments (1-6 weeks and 6-10 weeks) and whether they are moderated by the relaxation engagements. Simple slope analysis was also used here for the interpretation of the interactions.

**Results:**

Analysis revealed a significant decrease in stress symptoms (β=–.25; 95% CI –0.32 to –0.17; *P*<.001) during the period of 1-6 weeks of app use that was maintained during the period of 6-10 weeks. Breathing exercises significantly moderated the reduction in stress symptoms during the period of 1-6 weeks (β=–.07; 95% CI –0.13 to –0.01; *P*=.03), while engagement in digital video sessions did not moderate stress scores. Engagement in digital video sessions, as well as breathing exercises, significantly moderated the reduction in perceived sense of burden and lack of productivity during weeks 1-6 and remained stable during weeks 6-10 on both items.

**Conclusions:**

This study sheds light on the association between stress level reduction and specific components of engagement in a digital health app, breathing exercises, and cognitive behavioral therapy–based video sessions. Our findings provide a basis for further investigation of current and moderating factors that contribute to the personalization of digital intervention. In addition, results may aid in developing a more comprehensive understanding of how digital intervention tools work for mental health and for whom they are most effective.

## Introduction

Stress is defined as an emotional or physical reaction typically caused by an external trigger [1,2] and is known to be a high-prevalence global problem, negatively affecting mental and physical health [3]. According to Gallup 2019 Global Emotions Report, the percentage of the American population who experienced stress (55%) is one of the highest in the world, as compared to the global average of 35% [4,5]. Furthermore, it is reported that distress levels have only increased during the COVID-19 pandemic due to health concerns, altered everyday life routines [6], and the unpredictability of its future implications [3]. A recent US stress survey conducted, reports an elevated average reported stress level from prepandemic levels [7]. Moreover, an alarming proportion of adults reported that stress has an impact on their day-to-day functioning, with more than a quarter (27%) saying that most days they are so stressed they cannot function [7].

Stress has a major impact on managing daily life, as everyone experiences it in varying forms every day [8]. According to the American Psychological Association’s 2014 Work and Well-Being Survey, 31% of employed adults indicated that they felt tense or stressed out during the workday [9]. Several studies investigating the understanding of how employee health impacts work performance have found stress can cause burnout, absenteeism, and reduced efficiency and performance [10], which can negatively impact the organization [11]. Yet, some resources can be implemented to help meet the pressures and demands faced at work [12].

There is evidence of improvements in health status due to workplace wellness programs for mental health [13,14]. In the past decade, the delivery of digital interventions has become increasingly popular; these interventions target common mental health illnesses [15] within the workplace due to their easy implementation [16]. Web-based interventions are shown to offer several advantages that may overcome some of the limitations of face-to-face approaches [17] as they are accessible through any internet-connected device. Among the working population, these interventions could especially benefit those who do not seek regular mental health treatment because of negative perceptions of mental health needs in the workplace [14].

It is reported that a substantial proportion of adults with common mental disorders fail to receive treatment [18]. The barriers thought to impede appropriate mental health care seeking have been well documented in the literature [18,19]. Many patients experience accessibility issues such as prolonged wait times, cost, clinic location, and transportation [20]. Others report that the stigma associated with mental illness diagnosis is the main reason that decreases their willingness to seek treatment [19]. Thus, digital interventions have the potential to overcome many of the barriers associated with seeking and accessing mental health care [18] and to provide increased convenience for patients allowing them access to care at any time.

Furthermore, digital health solutions aim to foster patient empowerment [21,22] by encouraging them to be proactive in the care process. These interventions allow people the right guidance and support for self-management, consequently addressing their own health-related goals [23]. Digital solutions also enable users to be better informed about their health, share experiences, assess and monitor specified health states, reach treatment, and improve communication between them and health care professionals [24].

In recent years, there has been an increase in the delivery of digital therapeutics targeting common mental health conditions [25,26]. Depression and anxiety, which have the highest prevalence among mental health conditions, are at the forefront of these targeted programs [26]. Previous research has demonstrated the efficacy of web-based interventions on the prevention and management of these conditions [27,28] and these conditions were even reported as efficacious as traditional face-to-face treatments [29].

Most treatment provided in behavioral health apps uses module-based sessions and the teaching of coping and management strategies based on cognitive behavioral therapy (CBT) principles, problem-solving therapy, and psychoeducation [20,26]. The digital application of CBT is showing promising results in the treatment of depression and anxiety disorders [30]. Additional research suggests that a successful approach to addressing digital health interventions is through a multicomponent design [13], using features such as tailored messages, reporting of thoughts, feelings, or behaviors [31]; relaxations features [5]; integrated therapist contact and other supplementary worksheets and engagements [20].

Notwithstanding that stress itself is not defined as a disorder, it can lead to major psychological and physical implications that could place people at risk for illness [17,32]. Given that stress represents a major threat to public health, effective and scalable solutions to accommodate the demand for stress-management interventions are needed [17]. The concept of “stress management” refers to the emotional, psychological, and behavioral methods used to develop skills to manage and reduce stress [33]. Several types of stress management interventions have been reported to be effective at reducing stress levels, such as CBT, time management, relaxation, and meditation exercises [34]. However, applying these self-help skills to achieve improvement requires motivation and self-discipline.

One of the easiest methods for managing stress-related symptoms is deep breathing exercises [3]; these exercises have been proposed as first-line and supplemental treatments for emotional disorders [35]. Several studies reported that the use of different breathing techniques showed significant improvement in mood states and perceived stress [36,37] resulting in an effective improvement in the management of stress in daily life [36]. Slow and deep breathing can have a significant impact on reducing stress feelings and activating the relaxation response. This exercise is efficient as it reduces the ventilation in the dead space of the lungs and decreases the effect of stress and strain on the body by shifting the balance toward the parasympathetic system [38].

In recent years, the number of studies on digitally delivered stress management has been rising, yet the overall effect of specific formats of treatment delivery remains unclear [17]. Moreover, existing web-based stress management programs differ in various aspects such as type, content, and guidance, and this may influence their efficacy [17].

A recurring element described in the literature is video-based self-administrated intervention programs, which are mainly rooted in a CBT framework [39,40]. Previous studies reported that video-based CBT has shown effectiveness for anxiety, sexual pain, and insomnia [39-41]. However, these video-based programs have not yet been integrated into the treatment of stress in a digital form. This method is described as a third waveform of CBT [40], which is characterized by concepts such as acceptance and mindfulness that focus on the person’s relationship to thought and emotions [42].

In their meta-analysis review of digital mindfulness-based interventions, Spijkerman et al [43] suggested that these interventions can be effective in reducing perceived stress. Mindfulness is a complete therapy that can minimize stress levels and improve psychological well-being because it includes various components of relaxation, such as yoga, deep breathing techniques, and focusing attention [44]. However, despite the abundance of digital mindfulness-based interventions and considering the structured variability [45], it is presently unknown whether different components of these interventions are effective.

In summary, although there has been an established body of research evaluating the efficacy of mobile app platforms for stress management, there has been less focus on the effectiveness of engagement with specific components and relaxation strategies for stress reduction. The purpose of this study is to evaluate the effect of a relaxation engagement digital behavioral health app on perceived stress. Our primary hypothesis is that in users with moderate-to-high stress levels, improvements in stress scores will be observed during the initial weekly period of the intervention and will be followed by maintenance for several weeks after. We hypothesized that engagement with certain digital components would moderate the reduction in stress levels. Specifically, we hypothesized that breathing exercises and video sessions would moderate stress levels’ reduction. We also anticipated a significant reduction in the perceived sense of burden and perceived level of productivity ranking as these measures were designed to reflect a change in perceived stress levels over time.

## Methods

### Behavioral Health App

The Dario Health behavioral health app is essentially a modular tool delivering emotion-focused support designed to apply to a range of mental health conditions. Members generally have access to the Dario Health behavioral health platform as part of their employee or health plan benefits. A clinically based screening tool assesses each person’s needs and guides users to the most efficient support. The app provides several CBT programs including depression, anxiety, anger, stress, and substance use, and can be used as a self-guided intervention or with the help of a certified coach. The structure of each program includes conceptual videos, textual skills, breathing exercises, and monitoring progress tools. This study focuses on tracking cohorts of users with stress scores measured by their responses to the stress questionnaire.

The stress program begins with a psychology education module about stress, identifying symptoms, as well as how it presents itself physically, mentally, and emotionally. The program includes short CBT-based and empirically supported whiteboard educational videos, introducing skills such as understanding the impact of stress, coping strategies, identifying stress signs and symptoms, and stress-related behaviors. By increasing the awareness of the individuals’ thoughts and feelings and gradually explaining specific situations leading to stress, these modules provide a way to promote stress self-management. During these skills-based sessions, various techniques are introduced to assist with reducing stress such as relaxation exercises, breathing exercises, monitoring progress, and coaching. The content is delivered to engage the users and appealingly convey information. Relaxation exercises provide a digital experience including audio or video sessions of mindfulness, muscle relaxation techniques, and automatic thoughts and emotions regulation.

The breathing exercises are instructed as a short up to 5-minute audio guide; each one educates about a different technique, that is, progressive muscle relaxation, diaphragmatic breathing, or 4-7-8 breathing.

Coaching is introduced to users for support and assistance and people who choose to obtain coaching services via the app.

### Measures

Stress levels were assessed over 10 weeks using a stress questionnaire. The questionnaire that objectifies the degree of stress severity is a 5-item self-reported, in-app delivered questionnaire. Each of the 5 items is scored from 0 (not at all) to 3 (nearly every day; Table 1). The questionnaire aims to assess an individual’s level of stress and the factors contributing to it. The questionnaire items reflect perceived stress such as a sense of burden, lack of productivity, and symptoms of stress such as lack of sleep, body tension, and irritability. As a severity measure, scores range from 0 (absent of stress symptoms) to 15 (severe stress levels). Stress scores of ≤5, 6-10, and >10 represent low, moderate, and high stress levels, respectively. Internal reliability testing of the questionnaire demonstrated an acceptable internal reliability with a Cronbach α of 0.73 [46].

**Table 1:** The components in the stress questionnaire grading stress severity.

Item	Questionnaire	Scoring
1	Have you felt overwhelmed or stressed about important responsibilities?	0=“Not at all” 1=“Several days” 2=“More than half the days” 3=“Nearly every day”
2	What about feeling as though you were not as productive as you should've been?	0=“Not at all” 1=“Several days” 2=“More than half the days” 3=“Nearly every day”
3	Had trouble sleeping because your mind was racing or worrying?	0=“Not at all” 1=“Several days” 2=“More than half the days” 3=“Nearly every day”
4	Felt tension in your neck, back, or shoulders?	0=“Not at all” 1=“Several days” 2=“More than half the days” 3=“Nearly every day”
5	Felt noticeably more irritable, restless, or agitated than usual?	0=“Not at all” 1=“Several days” 2=“More than half the days” 3=“Nearly every day”

Independent variables were included in the analysis as potential moderating factors such as app sessions, videos, breathing exercises, and coaching interactions.

All data were transferred and stored in compliance with HIPAA (Health Insurance Portability and Accountability Act) requirements, using MongoDB or BigQuery database services. All data were anonymized before extraction for this study.

### Users

A retrospective data evaluation study was performed on the Dario database.

The stress cohort consisted of a group of 490 users who used the Dario Behavioral Health platform between 2019 and 2022. The population included users who started at a moderate and above level of stress (stress questionnaire score >5) and those who completed at least two stress assessments. The sample included 388 (79%) women, 95 (19%) men, and 7 (2%) others. The age distribution was as follows: ≤35 (38%), 36-55 (49%), 56-65 (12%), and 66-76 (1%) years. The average baseline score of the stress questionnaire was 9.8 (SD 2.7; median 10, IQR 4). The weekly average time of app use observed in this cohort was 26.4 minutes (SD 39.2; median 14, IQR 21)

### Ethical Considerations

All data used for the analysis were anonymized before extraction for this study. This study received an exemption from the institutional review board. Ethical & Independent Review Services [47], a professional review board, issued the institutional review board exemption for this study (21235 - 01#).

### Statistical Analysis

A classical linear longitudinal model assumes a single-slope growth pattern for changes in an outcome variable across time. In contrast, piecewise‐based mixed effects models allow flexibility in the modeling of variable change trajectories across time [48]. Previous studies consistently demonstrated a 2-stage dynamic of clinical outcomes associated with digital interventions: an initial improvement is followed by stabilization of the outcome [49,50].

Following a visualization of the distribution of the stress estimates over time, a piecewise cutoff point for the model slopes was chosen at 6 weeks of product use, assuming a change in the time-related stress severity level trajectory after 6 weeks considering previous research as well [51,52]. Such a behavior including 2 trajectories is in line with our previous research on digital follow-up measures in chronic conditions management [53,54].

Here, a piecewise mixed effects model was conducted to model the trajectory of the stress questionnaire mean score in 2 segments to allow the data to exhibit different linear trends over different periods. We tested whether digital engagement moderated 2 piecewise time trajectories in stress (1-6 weeks and 6-10 weeks). The model included a person-based random intercept and random slope for the time trajectory after the piecewise cutoff. Simple slope analysis was used for the interpretation of the interactions probing the moderators at 1 SD below (low engagement) and 1 SD above (high engagement) the average.

Next, we used mixed model analysis to model the trajectory of questions 1 and 2 rates (Table 1) in the stress questionnaire in the 2 segments (1-6 weeks and 6-10 weeks). Moreover, we tested whether these scores were moderated by engagement with specific components in the app. The models included a random intercept and random slope of the time trajectory. Simple slope analysis was used for the interpretation of the interactions probing the moderators at 1 SD below (low engagement) and 1 SD above (high engagement) the average.

## Results

### First Analysis: Stress Score Reduction Is Associated With Breathing Exercises

In total, 90% (441/490) of the users improved or maintained their stress level over this study’s period, that is, remained at a moderate level or improved from moderate or high to a low level of stress.

**Figure 1:**
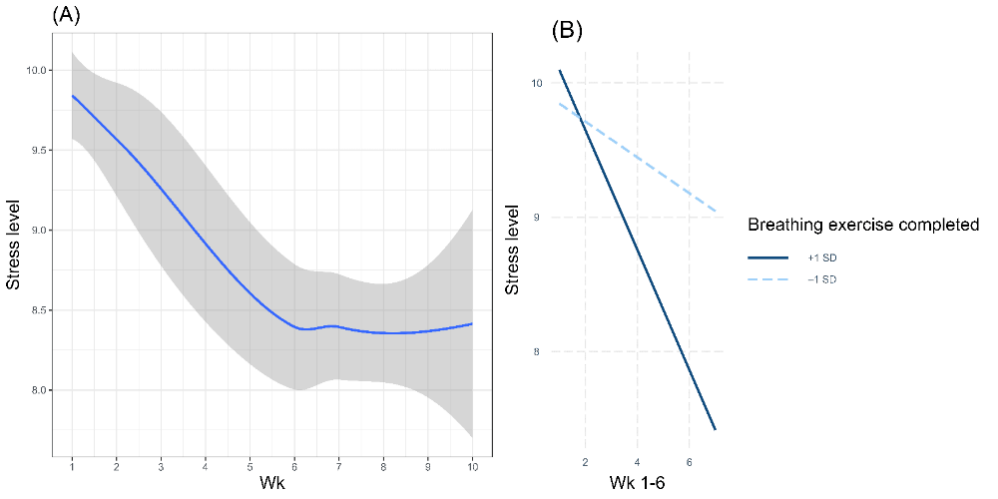
Stress level fluctuation over the period of 10 weeks of app use. (A) The blue line presents a smoothing of stress levels over time. The gray area around the line represents 95% CI. (B) The graph presents a simple slope analysis of the stress time trajectories during weeks 1-6 of app use moderated by the completed breathing exercise engagement.

Piecewise mixed model analysis revealed a significant decrease in stress levels (β=–.25; 95% CI –0.32 to –0.17; *P*<.001) during the period of weeks 1-6. There were no significant time-related trends in stress during the period of weeks 6-10 (β=.00; 95% CI –0.21 to 0.22; *P*=.98). Figure 1A demonstrates time-related fluctuations in stress levels.

Breathing exercises significantly moderated the reduction in stress score during the period of weeks 1-6 (β=–.07; 95% CI –0.13 to –0.01; *P*=.03). The weekly average number of breathing exercises completed is 1.55 (2.34 SD). Users with increased breathing exercise completion (+1 SD) demonstrated stronger reduction in stress score (β=–.45; 95% CI –0.64 to –0.25; *P*<.001) compared to users who completed fewer breathing exercises (–1 SD), that was not significant (β=–.13; 95% CI –0.32 to 0.05; *P*=.15). Breathing exercise did not moderate the time trajectory of stress levels during the period of weeks 6-10 (β=–.02; 95% CI –0.23 to 0.19; *P*=.87).

Figure 1B presents low and high levels of breathing exercise engagement, showing a significant reduction in stress levels only for users with high engagement, while the decrease for users with low engagement was not significant.

The engagement in digital video sessions did not significantly moderate stress score during both segments of time (β=–.01; 95% CI –0.03 to 0.00; *P*=.14 and β=–.03; 95% CI –0.11 to 0.04; *P*=.37 to week 1-6 and 6-10 accordingly).

### Second Analysis: Improvement in Perceived Stress Is Moderated by Digital Video Sessions

#### Overview

The perceived stress level was reflected by items 1 and 2 in the questionnaire.

#### Question 1: “Have you felt overwhelmed or stressed about important responsibilities?”

Piecewise mixed model analysis revealed that breathing exercises significantly moderated the reduction in question 1 ranking during the period of weeks 1-6 (β=–.02; 95% CI –0.04 to –0.01; *P*=.009). Breathing exercises did not moderate the time trajectory of question 1 ranking during weeks 6-10 (β=–.00; 95% CI –0.06 to 0.06; *P*=.91). The weekly average number of breathing exercises completion is 1.55 (2.34 SD). Users with increased breathing exercise engagement (+1 SD) demonstrated a stronger reduction in perceived stress prompted by important responsibilities (β=–.13; 95% CI –0.19 to –0.08; *P*<.001) compared to users who were less engaged in breathing exercises (–1 SD), that was not significant (β=–.02; 95% CI –0.07 to 0.03; *P*=.40).

Further piecewise mixed model analysis revealed that engagement in digital video sessions significantly moderated the reduction in question 1 ranking during the period of weeks 1-6 (β=–.01; 95% CI –0.01 to –0.00; *P*=.02), yet no significant reduction was seen during the period of 6-10 weeks (β=–.00; 95% CI –0.02 to 0.02; *P*=.91). The weekly average number of video session completion is 6.38 (8.59 SD). In addition, it was found that users who completed a higher number of video sessions (+1 SD) demonstrated a greater reduction in question 1 rank (β=–.13; 95% CI –0.19 to –0.07; *P*<.001) compared to those who were less engaged (–1 SD) with video sessions (β=–.02; 95% CI –0.08 to 0.03; *P*=.38).

#### Question 2: “What about feeling as though you were not as productive as you should’ve been?”

A piecewise mixed model has shown that the completion of breathing exercises has also significantly moderated the reduction of question 2 ranking in the stress questionnaire during the period of weeks 1-6 (β=–.02; 95% CI –0.04 to –0.00; *P*=.04); however, the period of 6-10 weeks was not significant (β=.01; 95% CI –0.05 to 0.07; *P*=.64). The weekly average number of breathing exercises completion is 1.55 (2.34 SD). Users with an increased number of completed breathing exercises (+1 SD) displayed a significant reduction in the question 2 ranking (β=–.07; 95% CI –0.13 to –0.01; *P*=.02), while in users who completed fewer breathing exercises (–1 SD), the reduction was not significant (β=.02; 95% CI –0.04 to 0.07; *P*=.55).

Here as well, an additional piecewise mixed model has demonstrated that engagement in video sessions significantly moderated the reduction in question 2 rank during weeks 1-6 (β=–.01; 95% CI –0.01 to –0.00; *P*=.01), yet no significant reduction was found in weeks 6-10 (β=.00; 95% CI –0.02 to 0.02; *P*=.84). The weekly average number of video session completion is 6.38 (8.59 SD). Furthermore, results from the simple slope analysis showed that users with increased engagement in video sessions (+1 SD) were significantly associated with improvement in question 2 rank (β=–.08; 95% CI –0.14 to –0.02, *P*=.01), compared to users with lower engagement (–1 SD) to video sessions (β=.03; 95% CI –0.02 to 0.08; *P*=.27).

## Discussion

### Principal Results

The Dario Health behavioral health stress program delivers an optimized solution that combines videos, textual skills, breathing exercises, and interaction with a coach to improve the users’ emotional wellness. The goal of this study was to assess changes in stress outcomes over time among users with moderate and above levels of stress scores at baseline. This study tested the effect of digital therapeutic use on stress levels over time, using a specific engagement instrument for stress conditions. Piecewise mixed model analysis indicated a significant decrease in stress levels during the period of the first 6 weeks of product use and maintained during the period from 6 to 10 weeks with no significant time-related trends. Engagement in breathing exercises significantly moderated the reduction in stress levels during weeks 1-6. During the period of 6-10 weeks, the reduced level of stress was maintained, and there were no significant time-related trends. An additional piecewise mixed model revealed that engagement in video sessions significantly moderated the reduction of perceived stress as shown by a decreased sense of burden (question 1) and a decreased lack of productivity (question 2), during weeks 1-6. It is worth noting that stress can have various causes and effects, and everyone experiences stress differently. However, a sense of burden and lack of productivity are commonly associated with stress, and identifying and acknowledging them can help individuals take steps to manage their stress levels and improve their well-being.

This real-world analysis presents new evidence regarding the dynamic efficacy of digital therapeutic solutions for people with moderate to high stress levels. The results are consistent with previous research that observed improvements in mental health metrics following digital, app-based interventions [26,29,33]. Our findings also provide insight into the nonlinear nature of stress level reduction during the first 10 weeks of a digital behavioral health program by showing the direct association with breathing exercises during the improvement stage in the first 6 weeks and emphasizing the key role of breathing exercises on stress reduction. In the next 6-10 weeks, the reduced levels of stress are maintained indicating that experiencing the use of a behavioral health digital program is a sustainable approach that may create a positive environment leading to a meaningful change. A nonlinear effect of digital therapeutics has previously been demonstrated in studies of mental health recovery via digital therapeutic intervention [50,55]. Additionally, the nonlinear effect of chronic condition management has been previously demonstrated in studies of chronic condition management such as glucose levels in diabetes, blood pressure, and pain level changes over time [53,54,56].

The study of nonlinear change requires multiple assessments over time and examination of the individual trajectories of variables [55,57]. If the variables of interest are assessed frequently over the course of product use, processes can be examined to better understand what facilitates and inhibits change. In this study, we examined time course data moving beyond the question of *whether* stress level change occurs and toward an understanding of *how* change occurs. A piecewise mixed model analysis indicated a significant decrease in stress level during the period of the first 6 weeks of product use and maintained during the period from 6 to 10 weeks with no significant time-related trends. This real-world intervention may have captured how change in coping may occur. In other words, people initially improve by learning to cope more effectively with stress but that needs to be incorporated, so this change can occur over only a limited period before it can be truly integrated. Based on current findings it appears that it takes 6 weeks to learn and then 6-10 weeks to integrate before becoming a changed behavior.

Although this real-world study does not relate to gender-specific experiences, it is notable that the gender ratio predominantly consists of women by 79% (388/490). Per previous studies, women are more likely to use e-mental health programs, designed features, and practices to reduce symptoms of stress or depression although they do not differ in terms of their general internet use [58]. The prevalence of major depressive episodes in women is about 2 times higher than that in men [59]. This could be explained by the existence of gender norms, men are less likely than women to disclose mental health symptoms and often delay seeking help until symptoms become severe [60,61]. From the social role perspective, women often have a multitasking agenda hence, efficiency and convenience of treatment may be more regarded [62,63].

In terms of physiological aspects, men and women were not seen to differ in terms of breathing practice and integrative body-mind training for dealing with stress conditions [64].

To date, there have been very few large-scale studies examining the effectiveness of digital therapeutic interventions for emotional disorders delivered in real-world settings [65-67]. There is also limited quantitative literature on the associations between the program components and direct methods with clinical outcomes [68].

We hypothesized that breathing techniques may be an enhanced method to address and reduce the bodily and mental processes associated with stress. Breathing exercises are an easily taught and practiced direct method, and they can realize almost immediate benefits and therefore allow for greater behavioral change. Stress causes sympathetic activation; the sympathetic nervous system is 1 of the 2 branches of the autonomic nervous system [35]. The sympathetic nervous system plays a critical role in the body’s response to stress. When it is activated, it triggers the “fight or flight” response, which prepares the body for immediate action in response to a perceived threat or danger [69]. The sympathetic nervous system is activated by the hypothalamus in response to a threat, which triggers the release of hormones such as adrenaline and noradrenaline [70]. Therefore, various physiological responses are caused such as increased heart rate, blood pressure, and respiratory rate, as well as the release of glucose from the liver to provide energy for the body’s response [71]. Our findings demonstrated that engagement with breathing exercises significantly moderated the reduction in stress levels during the period of weeks 1-6. Slow and deep breathing is known to activate the parasympathetic nervous system.

Stress can have various causes and effects, and everyone experiences stress differently. However, a perceived sense of burden and a perceived lack of productivity are commonly associated with stress. Identifying and acknowledging these stress-related feelings are the first steps in taking proactive measures to address them. Therefore, we also hypothesized that the ranking of the two items in the questionnaire, that is, (1) “Have you felt overwhelmed or stressed about important responsibilities?” and “(2) What about feeling as though you were not as productive as you should’ve been?” would be associated with engagement with certain digital components. We assumed that an improvement in perceived stress is reflected by the ways those 2 items would be linked to product use when combining video sessions with digital tools. Our findings showed that both items’ rankings 1 and 2 were moderated by engagement in video sessions as well as in breathing exercises. Engagement with CBT-based video sessions has the potential to be effective and efficient in treating symptoms of mental health conditions in real-world settings [72-74]. Managing stress and handling challenges are obtained by learning how to cope with stress in day-to-day life and adjusting it to the level of stress necessary for optimal functioning. The video sessions explain the core concepts of problem-solving and decision-making to reduce burden and modulate perceptions of responsibilities.

One of the strategies that helped to manage stress and improve well-being is setting realistic goals and prioritizing tasks [75]. Offering content and recommendations about breaking down tasks into manageable steps and setting realistic goals that combat the style of perfectionistic thinking possibly helped individuals to reduce feelings of burden and increase productivity reflected in items 1 and 2 in the questionnaire.

It was consistently demonstrated in our findings that breathing exercises moderated the improvement of the general stress score and the ranking of items 1 and 2 as well. Breathing exercises have a significant impact on reducing perceived stress and promoting relaxation [64,76]. Deep breathing exercises, such as diaphragmatic breathing or belly breathing, engage the diaphragm muscle and stimulate the parasympathetic nervous system. The parasympathetic nervous system is the other branch of the autonomic nervous system, and it is responsible for promoting rest, relaxation, and digestion [35]. It has been demonstrated that during respiration, inspiration inhibits sympathetic nervous system activity [77]. Therefore, breathing techniques could be used as first-line and supplemental treatments for stress and emotional disorders [35,78]. Deep breathing exercises can help slow down the heart rate, bringing it closer to a calm and regular rhythm, contributing to a feeling of relaxation [79]. By consciously focusing on deep breaths and consciously relaxing the muscles, breathing exercises help release tension and promote a sense of physical relaxation [79]. During breathing exercise oxygen supply is increased supporting better delivery of oxygen to the body’s tissues, including the brain [80]. It can improve mental clarity, focus, and overall well-being. Lastly, engaging in breathing exercises requires focusing attention on the breath. This helps shift the focus away from stressors, worries, and racing thoughts, promoting a mindful state of being in the present moment. Mindfulness has been shown to reduce stress and enhance overall psychological well-being [81].

Recent reviews in the field of mental health programs highlight the need for research that goes beyond examining the overall effects of such programs. Instead, there is a call for more nuanced investigations that delve into the associations between specific components or building blocks of these programs and the clinical outcomes observed. This approach aims to comprehend the relative contributions of various activities within digital health management protocols. Researchers and digital health organizations can gain a deeper understanding of what works best in digital health management protocols and improve the effectiveness of mental health interventions [65,68].

Our findings indicate that breathing exercises and CBT-based video sessions help people lower their stress levels. These findings suggest that increasing digital engagement to specific activities in a lower engaged population may be an efficient way to optimize digital platforms supporting patients with moderate levels of stress. New models of care and digital tools have the potential to transform health care by increasing accessibility, promoting patient-centered care, improving timeliness, enhancing equity, and optimizing efficiency. Digital tools can empower patients to take an active role in their health care journey. This shift toward patient-centered care promotes collaboration and empowers individuals to make informed choices about their health [82,83].

We expect that the analytical approach applied in this study will be beneficial for personalizing interventions and optimizing incentivization planning. This information could be used to further personalize outreach to encourage users to maintain their personal critical level of mental health activities. More research is required to further understand the current and potential moderating factors of digital intervention tools for mental health and how independent use of these tools facilitates the care of patients with day-to-day stress levels.

### Limitations

In this study, we used a single-arm design that analyzed retrospective real-world data that lacked a comparison group or control group, contained a self-selection bias, and lacked randomization. While this design allowed us to explore the association between digital relaxation tools and stress levels, we acknowledge the limitation of not having a control group. The lack of a control group restricts our ability to establish causal relationships and control potential confounding variables. Future research efforts could consider incorporating a control group to further validate and extend the implications of our findings. Additionally, we acknowledged the gender distribution predominantly consisting of 79% (388/490) women that may potentially influence the results and interpretation of the results. However, women have higher rates of anxiety and depression and use more digital treatment [58,59], therefore our results may be specifically applicable to the real world. In other words, the real-world population of women who have more anxiety and depression may be better served with the use of a mobile app. Although this study’s research topics are not related to gender-specific experiences, future research should strive for a more balanced gender representation. It is also possible that people who chose to use digital therapeutic instruments were those who were most motivated to make a change in their conditions. These factors could limit the generalizability and create a challenge for drawing causal conclusions. The time scale was designed to reflect a weekly interval change over 10 weeks; however, the relationships of interest in this study could be potentially investigated in different scales emphasizing daily or monthly outcomes. Owing to the difficulty in tracking daily changes in stress levels, most real-world studies focus on weekly fluctuations.

Stress level was the key outcome assessed in this study by using a stress questionnaire constructed by our internal experts. This questionnaire demonstrated a Cronbach α value of 0.73, an adequate reliability coefficient, as >0.70 is considered a measure of internal consistency [84,85]. It was decided to use our stress questionnaire although the Perceived Stress Scale-14, a 14-item scale, is the most widely used for measuring perceived stress [86] since it has fewer items and may be more useful in increasing the answer rate among platform users.

### Conclusions

This study provided evidence that an increase in mental health management via digital engagement, specifically with relaxation tools, can help reduce stress levels over time. Although stress can be experienced in various forms, it is established that using an app as an integrated part of the health self-management process significantly improves clinical outcomes. It was demonstrated that breathing exercises have a significant impact on promoting relaxation. From a behavioral perspective, engaging with CBT content helps recognize stress causes and provides strategies to self-manage the feelings of burden, ultimately improving well-being. These findings provide a basis for encouraging users to take an active role and make informed choices about their health. Future work is needed to investigate current and other potential moderating factors and to develop a more comprehensive understanding of the importance of the app, how digital intervention tools work for mental health, and for whom they are most effective. This knowledge will guide the development and implementation of personalized and evidence-based digital interventions, ultimately improving mental health outcomes for individuals in a variety of contexts.

## Abbreviations

CBT: cognitive behavioral therapy
HIPAA: Health Insurance Portability and Accountability Act
